# Premature Burial and the Law

**Published:** 1912-04-20

**Authors:** Jas. R. Williamson

**Affiliations:** 100 Chedington Road, Upper Edmonton, London


*To the Editor of* The Hospital.

### PREMATURE BURIAL AND THE LAW.

*To the Editor of* The Hospital.

Sir,—I had the pleasure of reading a very able and instructive article on "Cremation" in The Hospital of the 6th inst. With reference to it, may I venture to remark that the difficulties in the way of exhumation under our present laws are often ignored by shallow writers, who assume, apparently on the ground that we do not hear of premature burial being actually consummated in these islands, that such tragedies do not happen at all, or that they are very rare? How can it be possible to hear of such cases when the evidence is legally barred to us? Not more than two corpses in one hundred thousand are exhumed, the Home Secretary being the only authority (except, in certain cases, a coroner) who can order the disinterment of a body. Statements on the rarity of premature burial are not only unwarranted by the direct evidence available, but are in flagrant contradiction to the inference which the indirect evidence justifies. We know by numerous authenticated cases that living persons may be, and are, mistaken for dead; we know that, unless considerable care be taken, the consequences of such errors must be the burial of persons apparently dead, but actually alive; and, moreover, we know that not the slightest care is demanded by law, nor is, as a matter of common knowledge, usually taken to distinguish apparent from real death. A medical certificate of death is not even a legal requisite prior to interment, and some 9,000 burials in England and Wales, and a much larger proportion in Scotland and Ireland, take place without it. Where a death certificate is given it is almost always supplied upon the information of relatives or friends who have received no medical training, and without the giver of such certificate taking the trouble to view the supposed or alleged corpse, and verify the fact of death. I should be pleased to send any of your readers who desire to assist in obtaining amendment of the burial laws, so as to render premature burial impossible, pamphlets on the subject on receipt of a stamped addressed envelope. Thanking you for your kindness, I remain, Sir, your obedient servant,

100 Chedington Road, Jas. R. Williamson.

Upper Edmonton, London, April 12.

